# Supplementary material for: Magnetic and electronic phase transitions probed by nanomechanical resonators
Source: Nat Commun. 2020 Jun 1;11:2698. doi: 10.1038/s41467-020-16430-2 (PMC7264344; doi:10.1038/s41467-020-16430-2)
Supplement: Supplementary file 1 — Supplementary Information [file 41467_2020_16430_MOESM1_ESM.pdf]

SUPPLEMENTARY INFORMATION: Magnetic and electronic phase transitions probed by nanomechanical resonators

Makars Šiškins,[1,*] Martin Lee,[1,*] Samuel Mañas-Valero,[2] Eugenio Coronado,[2]
Yaroslav M. Blanter,[1] Herre S. J. van der Zant,[1] and Peter G. Steeneken[1,3,†]

[1] *Kavli Institute of Nanoscience, Delft University of Technology, Lorentzweg 1, 2628 CJ, Delft, The Netherlands*

[2] *Instituto de Ciencia Molecular (ICMol), Universitat de València, c/Catedrático José Beltrán 2, 46980 Paterna, Spain*

[3] *Department of Precision and Microsystems Engineering, Delft University of Technology, Mekelweg 2, 2628 CD, Delft, The Netherlands*

CONTENTS

Supplementary Note 1. Fundamental resonance frequency of a circular plate and membrane 1

Supplementary Note 2. Mechanical resonances and specific heat of MPS_3 (M=Fe, Ni, Mn) 3

Supplementary Note 3. Entropy in a suspended antiferromagnet 5

Supplementary Note 4. Dissipation and thermoelastic damping in vibrating membranes 6

Supplementary Note 5. Electric field induced strain in a circular $FePS_3$ membrane 7

Supplementary Note 6. Mechanical resonances and specific heat of 2H-TaS_2 near the charge density wave transition 8

Supplementary Note 7. Crystal growth and characterization 9

Supplementary Note 8. Reproducibility of measurements 10

Supplementary References 12

Supplementary Note 1. FUNDAMENTAL RESONANCE FREQUENCY OF A CIRCULAR PLATE AND MEMBRANE

In this section we analyze in more detail the resonance frequency of the $FePS_3$ resonators and show that near the phase transition they are close to the membrane limit. The fundamental resonance frequency of the mechanical resonator, f_0, in the crossover membrane-plate regime can be approximated as [1, 2]:

$$f_0(T) \approx \sqrt{f^2_{\text{membrane}} + f^2_{\text{plate}}} = \sqrt{\left(\frac{2.4048}{\pi d}\right)^2 \frac{E}{\rho}\frac{\epsilon(T)}{(1-\nu)} + \left(\frac{10.21t}{\pi d^2}\right)^2 \frac{E}{3\rho\,(1-\nu^2)}}, \tag{1}$$

where d is the diameter of the membrane, E the Young's modulus, $\epsilon(T)$ the strain, ν the Poisson's ratio, t the thickness, ρ the mass density and T the temperature. The resonance frequency of the fundamental mode of a circular

---

* These authors contributed equally.

† e-mail: p.g.steeneken@tudelft.nl; h.s.j.vanderzant@tudelft.nl; m.siskins-1@tudelft.nl

resonator is thickness dependent. For plate resonators $f_{\mathrm{plate}} \propto t$, as expected for small and linear deflection [1–3]. For membranes, however, f_0 is dominated by the biaxial tension N:

$$f_{\mathrm{membrane}} = \frac{2.4048}{\pi d}\sqrt{\frac{N}{\rho t}}. \tag{2}$$

Supplementary equation (2) yields $f_{\mathrm{membrane}} \propto t^{-0.5}$ for thin resonators. When the membrane is subjected to temperature changes, the total tension is dominated by thermal strains $\epsilon_{\mathrm{r}}^{\mathrm{th}}$. Thermal strains are of dilatational nature and do not cause any shear, thus, these can be written as: $\epsilon_{\mathrm{r}}^{\mathrm{th}} = \alpha \Delta T$, where α is the thermal expansion coefficient. In-plane radial thermal strain is then related to tension according to the Hooke's law as $N = N_0 + Et\epsilon_{\mathrm{r}}^{\mathrm{th}}/(1-\nu)$, where N_0 the intrinsic pre-tension introduced during the fabrication process. Thus, supplementary equation (1) is used to determine if the membranes under study are in the plate or in the membrane limit at a given temperature, as shown in Supplementary Fig. 1a,b.

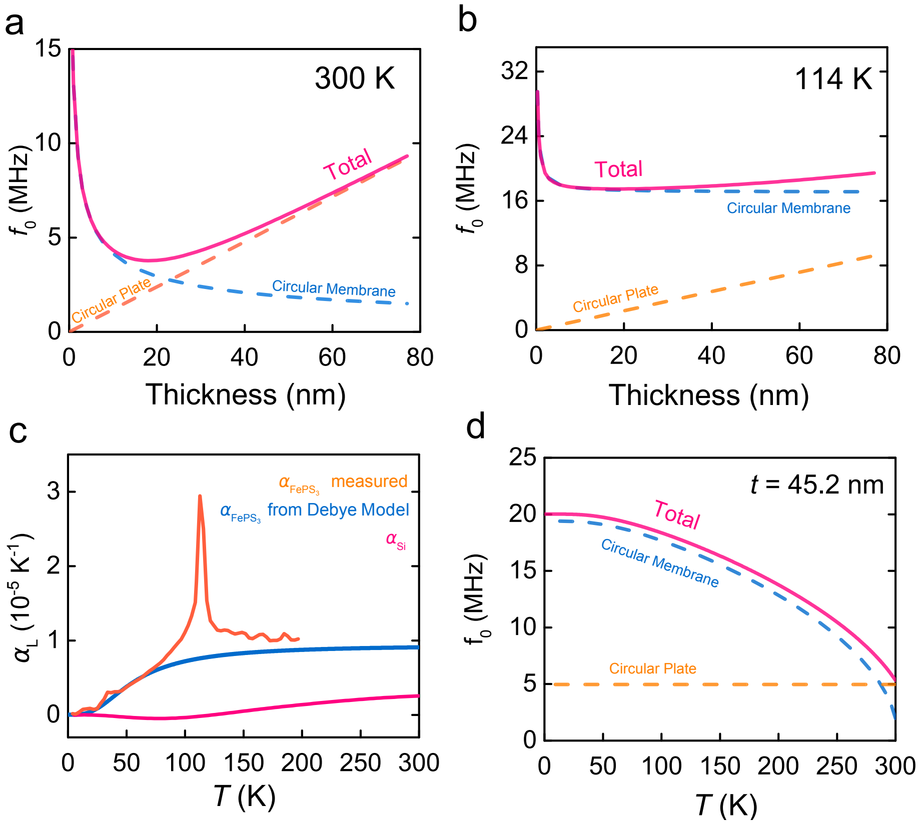

SUPPLEMENTARY FIG. 1. Resonance frequency of a $FePS_3$ resonator as a function of thickness for (a) 300 K and (b) 114 K. (c) Comparison of thermal expansion coefficients of $FePS_3$ as measured (solid orange line) and as predicted from the Debye model (solid blue line) to that of the Si substrate (solid magenta line). (d) Calculated resonance frequency of a $FePS_3$ membrane using supplementary equation (1) with a diameter of 10 μm taking into account the thermal expansion coefficient of $FePS_3$ as predicted by the Debye model.

The relative contribution of the plate term to the frequency of the resonator is largest at room temperature because the membrane tension is the lowest. As shown in Supplementary Fig. 1a at $T = 300$ K and $N_0 = 0.1$ N m^{-1}, thicker $FePS_3$ samples ($t > 40$ nm) behave as circular plates. However, as shown in Supplementary Fig. 1b, in proximity of the transition temperature ($T \approx 114$ K) due to temperature-induced strain $\epsilon(T)$ (see supplementary equation (1)), the resonator behaves close to the membrane limit over a thickness range from zero to 60 nm.

The total strain in the membrane is estimated using $\epsilon(T) = \epsilon_0 - \int_{300\mathrm{K}}^{T}(\alpha_{\mathrm{material}}(T) - \alpha_{\mathrm{Si}}(T))\mathrm{d}T$, where ϵ_0 is the intrinsic pre-strain at $T = 300$ K [4, 5]. Because $\alpha_{\mathrm{SiO_2}} \ll \alpha_{\mathrm{Si}}$ [6, 7], the effect of the thin SiO_2 layer can be neglected. As shown in Supplementary Fig. 1c, the thermal expansion coefficient of the silicon substrate (solid magenta line) is small compared to that of $FePS_3$. Therefore, the total strain in the membrane will mainly build up due to $\alpha_{\mathrm{FePS_3}}$, and this term dominates the change in resonance frequency, $f_0(T)$ as depicted in Supplementary Fig. 1d.

Supplementary Note 2. MECHANICAL RESONANCES AND SPECIFIC HEAT OF MPS_3 (M=Fe, Ni, Mn)

In addition to $FePS_3$, we measure $MnPS_3$ and $NiPS_3$ membranes with a 10 μm diameter and thicknesses of 31.8 ± 1.2 and 35.7 ± 1.1 nm, respectively. Comparative study of these is particularly interesting since $FePS_3$ is an Ising antiferromagnet, while the other two are Heisenberg ($MnPS_3$) and XY ($NiPS_3$) antiferromagnets. The resonance peak of the fundamental membrane mode, $f_0(T)$, as well as the Q-factor is measured from $4-200$ K using the procedure described in the main text. We note that the resonance frequency and quality factor are related via causality: $f(T) = f_0(T)\sqrt{1 - \frac{1}{4Q(T)^2}}$. However, for a quantitative change in the Q-factor next to the phase transition in samples presented in Fig. 2c of the main text and Supplementary Fig. 2, we expect the effect on $f_0(T)$ to be of the order of kHz, which is insignificant compared to the change in frequency up to ~ 2 MHz.

At the phase transition, significant changes in both the resonance frequency (Supplementary Fig. 2a) and quality

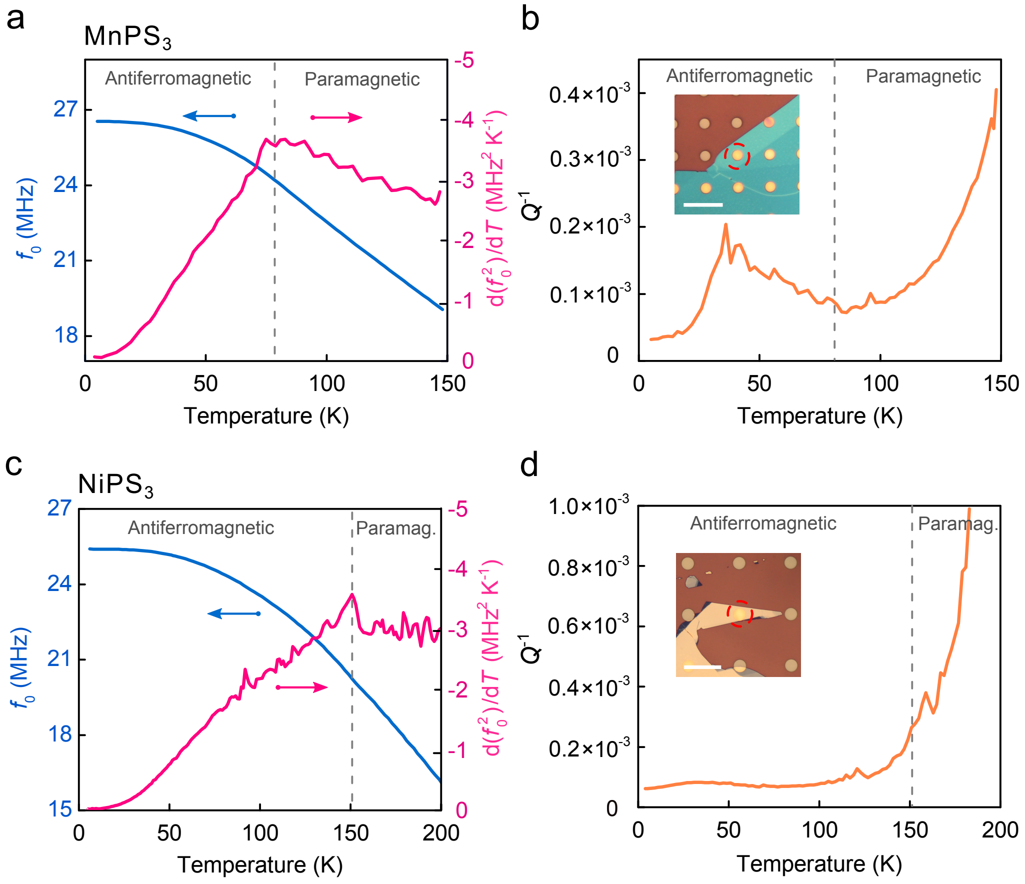

SUPPLEMENTARY FIG. 2. Mechanical properties of $MnPS_3$ and $NiPS_3$ membranes. Dashed vertical lines indicate transition temperatures, T_N. (a) Solid blue line - Measured resonance frequency of $MnPS_3$ membrane as a function of temperature. Solid magenta line - Temperature derivative of f_0^2. (b) Measured mechanical damping (Q^{-1}) as a function temperature. Inset: Optical image of the sample, $t = 32.3 \pm 0.4$ nm. Scale bar: 40 μm. (c) Solid blue line - Measured resonance frequency of $NiPS_3$ membrane as a function of temperature. Solid magenta line - Temperature derivative of f_0^2. (b) Measured mechanical damping (Q^{-1}) as a function temperature. Inset: Optical image of the sample, $t = 35.7 \pm 0.5$ nm. Scale bar: 30 μm.

factor (Supplementary Fig. 2b) of the $MnPS_3$ membrane are observed. Supplementary figure 2a shows the resonance frequency (solid blue line) and the corresponding $\frac{\mathrm{d}(f_0^2)}{\mathrm{d}T}$ (solid magenta line) with a peak that occurs at a temperature similar to the transition temperature from the antiferromagnetic phase (< 78 K) to the paramagnetic phase (> 78 K) in the bulk material [8] and is thus attributed as T_N. The temperature dependence of $\mathrm{d}(f_0^2)/\mathrm{d}T$ shows a broad hump with a smeared peak at T_N, which resembles the temperature dependent specific heat $c_v(T)$ of this material in bulk form [8]. Supplementary figure 2b shows the mechanical dissipation, Q^{-1}, that exhibits a local minimum close to T_N as well as a local maximum at $T \sim 36$ K. In Supplementary Fig. 2c the resonance frequency of the $NiPS_3$ membrane (solid blue line) is shown with the corresponding $\frac{\mathrm{d}(f_0^2)}{\mathrm{d}T}$ (solid magenta line). A small peak is noticeable in $\frac{\mathrm{d}(f_0^2)}{\mathrm{d}T}$ near

bulk $T_N \sim 155$ K indicating the phase transition [9]. However, as shown in Supplementary Fig. 2d, no significant anomalies in the Q-factor were observed in the case of $NiPS_3$. Compared to $FePS_3$, the effect of the phase transitions in $MnPS_3$ and $NiPS_3$ on the resonances is more gradual (Supplementary Fig. 2a-d). Both materials show a peak in $\frac{d(f_0^2)}{dT}$ at the T_N, but their dissipation does not show a clear jump at T_N like in the case of $FePS_3$.

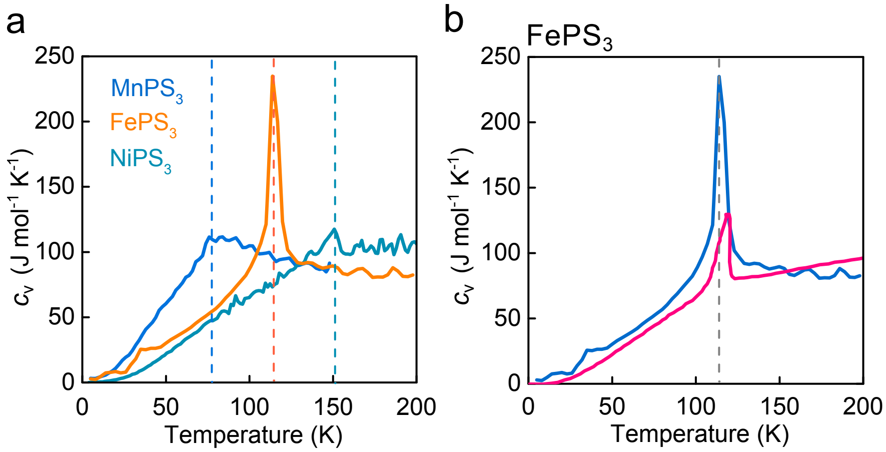

SUPPLEMENTARY FIG. 3. Estimated specific heat (c_v) for (a) MPS_3 (M=Fe, Mn, Ni) membranes of $t = 45.2 \pm 0.6$ nm, 31.8 ± 1.2 nm and 35.7 ± 1.1 nm, respectively. Dashed lines indicate the corresponding transition temperatures (T_N): $T_N = 76 \pm 5$ K for $MnPS_3$, $T_N = 114 \pm 3$ K for $FePS_3$ and $T_N = 151 \pm 5$ K for $NiPS_3$. (b) Specific heat of ultrathin $FePS_3$ membrane compared to that of its bulk form. Solid blue line - measured c_v of the $FePS_3$ ultrathin membrane. Solid magenta line - c_v reported by Takano et al. [8] for a bulk crystal.

We also calculated the corresponding temperature dependent specific heat $c_v(T)$ for the three MPS_3 samples. Following the methodology described in the main text, we estimate the Grüneisen parameter following the Belomestnykh−Tesleva relation [10, 11]: $\gamma \approx \frac{3}{2}\left(\frac{1+\nu}{2-3\nu}\right)$. We use reported values for monolayers of $FePS_3$ ($C_{11} = 72.7$ N m^{-1}, $C_{12} = 22.1$ N m^{-1} and $\rho_{2D} = 2.16 \times 10^{-6}$ kg m^{-2}), $MnPS_3$ ($C_{11} = 61.7$ N m^{-1}, $C_{12} = 20.2$ N m^{-1} and $\rho_{2D} = 2.00 \times 10^{-6}$ kg m^{-2}) and $NiPS_3$ ($C_{11} = 87.0$ N m^{-1}, $C_{12} = 23.1$ N m^{-1} and $\rho_{2D} = 2.15 \times 10^{-6}$ kg m^{-2}) as obtained from first-principles calculations [12, 13]. We convert these to three-dimentional Young's modulus E, mass density ρ and Poisson's ratio ν using $E = \frac{C_{11}^2 - C_{12}^2}{C_{11}}\frac{1}{t}$, $\rho = \frac{\rho_{2D}}{t}$ and $\nu = \frac{C_{12}}{C_{11}}$, assuming the interlayer distance for the compounds to be $t_{FePS_3} = 0.64$ nm, $t_{MnPS_3} = 0.65$ nm and $t_{NiPS_3} = 0.64$ nm as determined from Supplementary Fig. 7a-c. The resulting values are $E = 103$ GPa, $\nu = 0.304$ and $\rho = 3375$ kg m^{-3} for $FePS_3$, $E = 85$ GPa, $\nu = 0.327$ and $\rho = 3076$ kg m^{-3} for $MnPS_3$ and $E = 126$ GPa, $\nu = 0.265$ and $\rho = 3359$ kg m^{-3} for $NiPS_3$.

In Supplementary Fig. 3a the specific heat for the three MPS_3 are displayed, as determined from the data in Supplementary Fig. 2a,c, and Fig. 2a using equation (2) from the main text. Detected temperatures are indicated by dashed lines and found to be $T_N = 76 \pm 5$ K for $MnPS_3$, $T_N = 114 \pm 3$ K for $FePS_3$ and $T_N = 151 \pm 5$ K for $NiPS_3$, which are in agreement with experiments in bulk crystals of MPS_3 [8, 14]. As shown in Supplementary Fig. 3b, for $FePS_3$ we obtain a good correspondence to bulk literature values [8] (solid magenta curve) without fitting parameters. The transition related peak in c_v, however, is more pronounced in the case of the ultrathin membrane (solid blue curve) than in the bulk.

Supplementary Note 3. ENTROPY IN A SUSPENDED ANTIFERROMAGNET

A uniaxial antiferromagnet with two antiparallel magnetic sub-lattices close to the phase transition can be modeled by the Landau theory of phase transitions [15, 16]. In this theory, the vector order parameter $\mathbf{L}$ is defined as the difference between the magnetizations of the two sublattices, $\mathbf{M}_1$ and $\mathbf{M}_2$ (thus, the antiferromagnetic vector $\mathbf{L} = \mathbf{M}_1 - \mathbf{M}_2$). This order parameter is zero in the paramagnetic phase and is finite in the antiferromagnetic phase. The magnetization, $\mathbf{M}$, is the sum of both magnetizations ($\mathbf{M} = \mathbf{M}_1 + \mathbf{M}_2$) and equals to zero in the absence of an external magnetic field, $\mathbf{H}$.

For a uniaxial crystal antiferromagnet, the transition temperature is commonly known as Néel temperature, T_N, but, as conveyed by Landau et al. [15, 16], it is also referred to as the antiferromagnetic Curie temperature and denoted as T_c. Note that further we will consider the Néel temperature, denoted as T_N, as the temperature where the transition from a paramagnetic to an antiferromagnetic phase takes place. Near T_N, $\mathbf{L}$ is small and the free energy, F, can be expanded in terms of $\mathbf{L}$ and $\mathbf{H}$, since the magnetization is only non-zero when an external field $\mathbf{H}$ is present (i.e. a spin-flop transition). Following Landau formalism and considering z-axis as the main axis of symmetry, we write (see e.g. Ref. 16):

$$F = F_0 + AL^2 + BL^4 + D\left(\mathbf{H}\cdot\mathbf{L}\right)^2 + D'H^2L^2 - \frac{1}{2}\chi_\mathrm{p}H^2 + \frac{1}{2}\beta\left(L_x^2 + L_y^2\right) - \frac{1}{2}\mu_\mathrm{M}\left(H_x^2 + H_y^2\right) - \frac{H^2}{8\pi}\ , \qquad (3)$$

where $A = a(T - T_\mathrm{N})$, D, D', a and B are phenomenological positive constants which are taken temperature independent, χ_p the isotropic susceptibility for $T > T_\mathrm{N}$, β the index that describes the temperature dependence of the spontaneous magnetization below T_N ($\beta > 0$ for $\mathbf{L}$ directed out-of-plane) and μ_M the magnetic susceptibility in the paramagnetic phase. The minimization of supplementary equation (3), where the vector $\mathbf{L}$ is along the z-axis (thus, $L_x = L_y = 0$ and $\beta > 0$) and in the absence of field ($H = 0$), gives $L_x = L_y = 0$ and $L_z = 0$ for $T > T_\mathrm{N}$ (paramagnetic phase) and $L_z = \sqrt{a(T_\mathrm{N} - T)/(2B)}$ for $T < T_\mathrm{N}$ (antiferromagnetic phase).

Now we introduce strain. For an easy-axis antiferromagnet near T_N with the vector $\mathbf{L}$ along the z-axis and in the absence of field, we can write supplementary equation (3) as:

$$F = F_0 + AL^2 + BL^4 + \frac{1}{2}\beta\left(L_x^2 + L_y^2\right) + \zeta L_z^2 + \zeta_x L_x^2 + \zeta_y L_y^2\ , \qquad (4)$$

where the last three added terms describe the magnetostriction effects, i.e., the coupling of magnetic moments to strain; the coefficients ζ, $\zeta_{x,y}$ are linear combinations of the components of the strain tensor. We assume that the strain is determined by the deformation of the membrane, and that the back-action exerted by the magnetization on the strain is negligible. In that case, for the calculation of the order parameter, ζ and $\zeta_{x,y}$ can be treated as temperature-independent constants. We also assume that $|\zeta|, |\zeta_{x,y}| \ll \beta$, so that even the strained antiferromagnet exhibits an easy-axis. The minimization of supplementary equation (4) gives $L_x = L_y = 0$, leading to a free energy which only depends on L_z,

$$F = F_0 + \left[a\left(T - T_\mathrm{N}\right) + \zeta\right]L_z^2 + BL_z^4. \qquad (5)$$

The first observation is that the magnetostriction effects shift the antiferromagnetic phase transition point. Indeed, the phase transition occurs at the temperature T_N^* at which the coefficient multiplying L_z^2 in supplementary equation (5) vanishes. This gives $T_\mathrm{N}^* = T_\mathrm{N} - \zeta/a$ (which is equation (4) in the main text).

Second, we calculate the behavior of the specific heat close to the phase transition. Minimizing supplementary equation (5) with respect to L_z, we find the equilibrium free energy, $F_\mathrm{min} = F_0 - a^2(T - T_\mathrm{N}^*)^2/(4B)$ in the antiferromagnetic phase, where F_0 is the free energy of the paramagnetic phase. We proceed by calculating the entropy $S_\mathrm{min} = -\partial F_\mathrm{min}/\partial T$,

$$S_\mathrm{min} - S_0 = \begin{cases} -a^2(T_\mathrm{N}^* - T)/(2B) & T < T_\mathrm{N}^* \\ 0 & T > T_\mathrm{N}^* \end{cases}\ , \qquad (6)$$

where $S_0 = -\partial F_0/\partial T$ is the entropy of the paramagnet with S_0 the non-magnetic contribution to the entropy. Since the renormalized transition temperature T_N^* is strain-dependent, the entropy of the antiferromagnet contains an additional (as compared to the paramagnetic phase) strain-dependent term. The specific heat near T_N^*, $c_\mathrm{v,min} = T\partial S_\mathrm{min}/\partial T$, reads

$$c_\mathrm{v,min} - c_\mathrm{v,0} = \begin{cases} T_\mathrm{N}^* a^2/(2B) & T < T_\mathrm{N}^* \\ 0 & T > T_\mathrm{N}^* \end{cases}\ , \qquad (7)$$

where $c_\mathrm{v,0}$ is the non-magnetic contribution to the specific heat. This derivation shows, in line with Landau theory [15, 16], that the specific heat has a jump at the transition temperature, T_N^*.

Supplementary Note 4. DISSIPATION AND THERMOELASTIC DAMPING IN VIBRATING MEMBRANES

For a membrane in motion the dissipation Q^{-1} is defined as the ratio of the energy lost per cycle to 2π times the stored energy. The total dissipation in a membrane is given by the sum of all contributing dissipation mechanisms [17]:

$$Q^{-1} = Q^{-1}_{\mathrm{medium}} + Q^{-1}_{\mathrm{clamping}} + Q^{-1}_{\mathrm{intrinsic}} + Q^{-1}_{\mathrm{other}}. \tag{8}$$

Q^{-1}_{medium} is related to losses due to the interaction with a fluid medium or gas and can thus be neglected in high vacuum. $Q^{-1}_{\mathrm{clamping}}$ is related to the transfer of mechanical energy to the anchoring substrate, which has a small temperature dependence [17]. $Q^{-1}_{\mathrm{intrinsic}}$ quantifies all intrinsic damping mechanisms of the material, such as thermoelastic damping, internal and surface friction, and phonon-phonon interaction loss. Q^{-1}_{other} relates all other possible damping mechanisms, such as electrical charge damping and magneto-motive damping. In this section we focus on deriving the expression for the thermoelastic damping. Further derivation in this section follows Zener's standard linear solid model [18, 19].

The dissipation is equal to the ratio between the imaginary and real parts of the complex elastic modulus $E^{*}(\omega) = E'(\omega) + \mathrm{i}E''(\omega)$:

$$Q^{-1} = \frac{E''}{E'}. \tag{9}$$

For a standard linear solid with a single relaxation mechanism, real and imaginary parts of the complex elastic modulus are given by:

$$E'(\omega) = E_{\mathrm{r}} + (E_{\mathrm{u}} - E_{\mathrm{r}})\frac{\omega^2\tau^2}{1+(\omega\tau)^2},\; E''(\omega) = (E_{\mathrm{u}} - E_{\mathrm{r}})\frac{\omega\tau}{1+(\omega\tau)^2}, \tag{10}$$

where ω is the resonance eigenfrequency, τ the thermal relaxation time, E_{r} and E_{u} are the relaxed (or isothermal) and unrelaxed (or adiabatic) Young's moduli, respectively (see Ref. 17 and 19 for more details). From supplementary equation (9), the dissipation is then:

$$Q^{-1} = (E_{\mathrm{u}} - E_{\mathrm{r}})\frac{\omega\tau}{E_{\mathrm{r}} + E_{\mathrm{u}}(\omega\tau)^2} \approx \frac{E_{\mathrm{u}} - E_{\mathrm{r}}}{E_{\mathrm{r}}}\frac{\omega\tau}{1+(\omega\tau)^2}, \tag{11}$$

for $(E_{\mathrm{u}} - E_{\mathrm{r}}) \ll E_{\mathrm{r}} \approx E_{\mathrm{u}}$. For a standard thermoelastic solid and in the case of thermoelastic damping Q^{-1}_{TED}, supplementary equation (11) can be rewritten as [18–20]:

$$Q^{-1}_{\mathrm{TED}} = \frac{E_{\mathrm{u}} - E_{\mathrm{r}}}{E_{\mathrm{r}}}\beta = \frac{E\alpha^2 T}{c_{\mathrm{v}}}\beta, \tag{12}$$

where α is the thermal expansion coefficient, c_{v} the specific heat and β the thermomechanical parameter, that in Zener's model is $\beta_{\mathrm{Z}} = \frac{\omega\tau}{1+(\omega\tau)^2}$. The exact expression for thermoelastic damping and its relation to the thermal properties of solids was found by Lifshitz and Roukes [20] with $\beta_{\mathrm{LR}} = \frac{6}{\xi^2} - \frac{6}{\xi^3}\frac{\sinh(\xi)+\sin(\xi)}{\cosh(\xi)+\cos(\xi)}$, where $\xi = \frac{\pi}{\sqrt{2}}\sqrt{\omega\tau}$. Under the assumption that the temperature dependence of $\omega\tau$ is small, supplementary equation (12) can be written as:

$$Q^{-1}_{\mathrm{TED}} \propto \frac{E\alpha^2 T}{c_{\mathrm{v}}}. \tag{13}$$

The close correspondence between this expression and the data in Fig. 2d and 4d in the main text indicates that this assumption is reasonable.
As could be noted from equation (2) in the main text and assuming the elastic properties of the material and its Grüneisen parameter to have a negligible temperature dependence at low temperatures, the thermoelastic damping Q^{-1}_{TED} is related to the frequency f_0 of the resonator as:

$$Q^{-1}_{\mathrm{TED}}(T) \propto \frac{E\alpha^2(T)T}{c_{\mathrm{v}}(T)} \propto c_{\mathrm{v}}(T)\,T \propto -T\frac{\mathrm{d}[f_0^2(T)]}{\mathrm{d}T}, \tag{14}$$

where we use $\alpha(T) \propto c_{\mathrm{v}}(T)$ and $c_{\mathrm{v}}(T) \propto \frac{\mathrm{d}[f_0^2(T)]}{\mathrm{d}T}$ according to equation (2) in the main text. Therefore, in accordance with our observations in the main text, an anomaly (a jump) in the specific heat at the transition temperature will be visible in both $\frac{\mathrm{d}[f_0^2(T)]}{\mathrm{d}T}$ and $Q^{-1}(T)$ if the thermoelastic damping is the dominating dissipation mechanism.

Supplementary Note 5. ELECTRIC FIELD INDUCED STRAIN IN A CIRCULAR $FePS_3$ MEMBRANE

A constant electrostatic load is applied to the circular membrane with a radius a (see Supplementary Fig. 4). This results in a uniform curvature R with a maximum deflection δ.

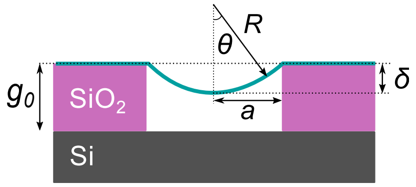

SUPPLEMENTARY FIG. 4. Schematic of the membrane deformation, cross-section view.

In this system, the electrostatic load is balanced by the total tension N_{total}:

$$\frac{\varepsilon_0 (V_{\mathrm{G}}^{\mathrm{DC}})^2}{2(g_0-\delta)^2}\pi R^2 = 2\pi N_{\mathrm{total}} R, \tag{15}$$

where ε_0 is the vacuum permittivity, $V_{\mathrm{G}}^{\mathrm{DC}}$ the applied voltage, g_0 the gap size between the membrane and the bottom silicon plate. For small deflections the radius of curvature can be approximated as:

$$R \approx \frac{a^2}{2\delta}, \tag{16}$$

so that the tension N_{total} becomes:

$$N_{\mathrm{total}} = \frac{\varepsilon_0 (V_{\mathrm{G}}^{\mathrm{DC}})^2}{2(g_0-\delta)^2}\frac{a^2}{4\delta}. \tag{17}$$

The radial strain in such a membrane can be estimated from the arc length [21, 22]:

$$\epsilon = \frac{R\theta - a}{a} \approx \frac{a^2}{6R^2}. \tag{18}$$

Combining this result with supplementary equation (16) yields:

$$\epsilon \approx \frac{2\delta^2}{3a^2}. \tag{19}$$

From Hooke's law and including supplementary equation (19), one can write the strain due to deformation as:

$$N = \frac{Et}{1-\nu}\epsilon = \frac{2Et\delta^2}{3a^2(1-\nu)}, \tag{20}$$

Therefore, the total tension in the membrane, including the thermal expansion induced tension $N_0(T)$ at a certain temperature T, can be written as:

$$N_{\mathrm{total}} = N_0(T) + N = N_0(T) + \frac{2Et\delta^2}{3a^2(1-\nu)} = \frac{\varepsilon_0 (V_{\mathrm{G}}^{\mathrm{DC}})^2}{2(g_0-\delta)^2}\frac{a^2}{4\delta}, \tag{21}$$

which also can be rewritten as:

$$V_{\mathrm{G}}^{\mathrm{DC}} = \sqrt{\frac{2(g_0-\delta)^2}{\varepsilon_0}\left(\frac{4\delta N_0(T)}{a^2} + \frac{8Et\delta^3}{3a^4(1-\nu)}\right)}. \tag{22}$$

As shown in Supplementary Fig. 5a, supplementary equation (22) in combination with (19) and (1) fits the experimental data well and is used to provide an an estimate of the electrostatically induced strain in the membrane (see Supplementary Fig. 5b).

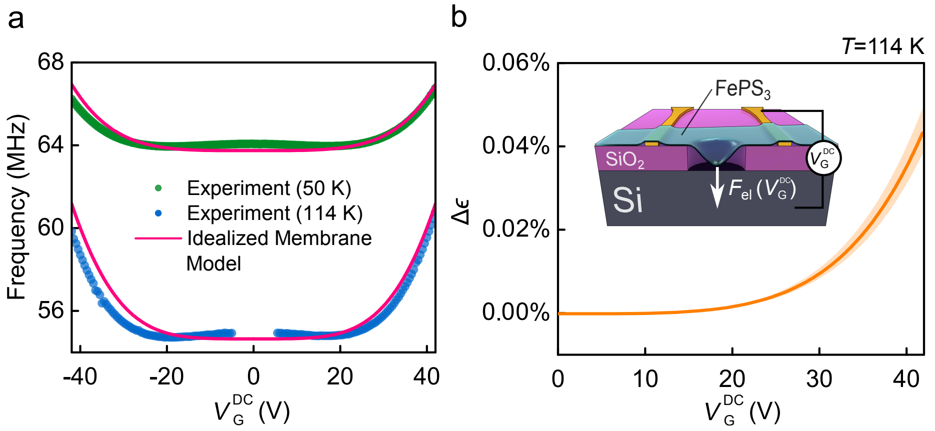

SUPPLEMENTARY FIG. 5. (a) Idealized electrostatically strained membrane model for $N_0 = 0.01$ N m^{-1}, $g_0 = 285$ nm, $a = 2$ μm, $t = 8$ nm, $E = 103$ GPa and $\nu = 0.304$; superimposed with corresponding experimental data. (b) Estimate of added radial strain at $T = 114$ K. The shaded orange region represents an estimated uncertainty from the accuracy in determining the temperature induced strain ($\pm 0.015\%$) at $T = 114$ K. Inset - schematics of the experiment.

Supplementary Note 6. MECHANICAL RESONANCES AND SPECIFIC HEAT OF 2H-TaS$_2$ NEAR THE CHARGE DENSITY WAVE TRANSITION

For 2H-TaS$_2$ resonance frequency measurements were performed on a $d = 4$ μm drum made of a 31 nm thin flake (see Supplementary Fig. 6a, solid blue line). As shown by the solid green line in Supplementary Fig. 6a, the specific heat-related temperature derivative of f_0^2 reveals a clear peak at $T_{\mathrm{CDW}} \sim 75$ K. We convert the measured $\frac{\mathrm{d}(f_0^2)}{\mathrm{d}T}$ to the specific heat using the methodology described in the main text. We use reported values of $E_{\mathrm{2D}} = 87$ N m^{-1} and $\nu = 0.27$ for a monolayer of 1H-TaS$_2$ obtained from molecular dynamics simulations [23] and mass density $\rho = 6110$ kg m^{-3}. We find the corresponding $E = E_{\mathrm{2D}}/t = 149$ GPa, taking the interlayer spacing $t = 0.58$ nm. The estimated specific heat of the 2H-TaS$_2$ membrane is depicted in Supplementary Fig. 6b (solid blue line). The four-probe resistance was measured on the same flake to confirm the existence of a CDW transition using a conventional electronic based method as shown in Supplementary Fig. 6c. The expected characteristic kink in the resistance is visible at ~ 77 K, consistent with the CDW transition temperature previously reported in 2H-TaS$_2$ [24].

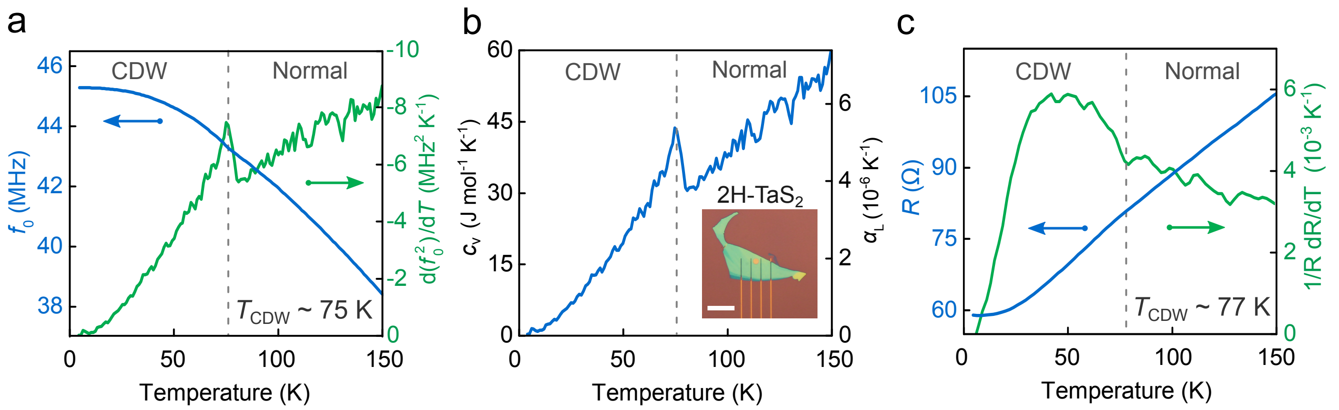

SUPPLEMENTARY FIG. 6. Mechanical properties of a 2H-TaS$_2$ resonator with membrane thickness of 31.2 ± 0.6 nm. Dashed vertical lines in the panels indicate the transition temperature, T_{CDW}. (a) Solid blue line - resonance frequency as a function of temperature. Solid green line - temperature derivative of f_0^2. (b) Solid blue line - estimated specific heat (c_{v}) and thermal expansion coefficient (α_{L}). Inset: optical image of the sample. Scale bar: 20 μm. (c) Solid blue line - four-point resistance of the same sample. Solid green line - $\frac{1}{R}\frac{dR}{dT}$ plot showing the CDW related feature at T_{CDW}.

Supplementary Note 7. CRYSTAL GROWTH AND CHARACTERIZATION

The crystal growth of MPS_3 (M = Mn, Fe, Ni) was performed following a typical solid state reaction. Powders of Mn (> 99.9%, from Sigma-Aldrich), Fe (99.998%, from Alfa-Aesar), Ni (99.99%, from Sigma-Aldrich), P (> 99.99%, from Sigma-Aldrich) and S (99.998%, from Sigma-Aldrich) were mixed in a stoichiometric ratio, pressed into a pellet and sealed in an evacuated quartz ampoule ($P \sim 5 \times 10^{-5}$ mbar, length = 25 cm, internal diameter = 1.5 cm) and heated from room temperature to 400 °C at 1.1 °C per minute. Then, the temperature was kept constant for twenty days and slowly cooled down (0.07 °C per minute).

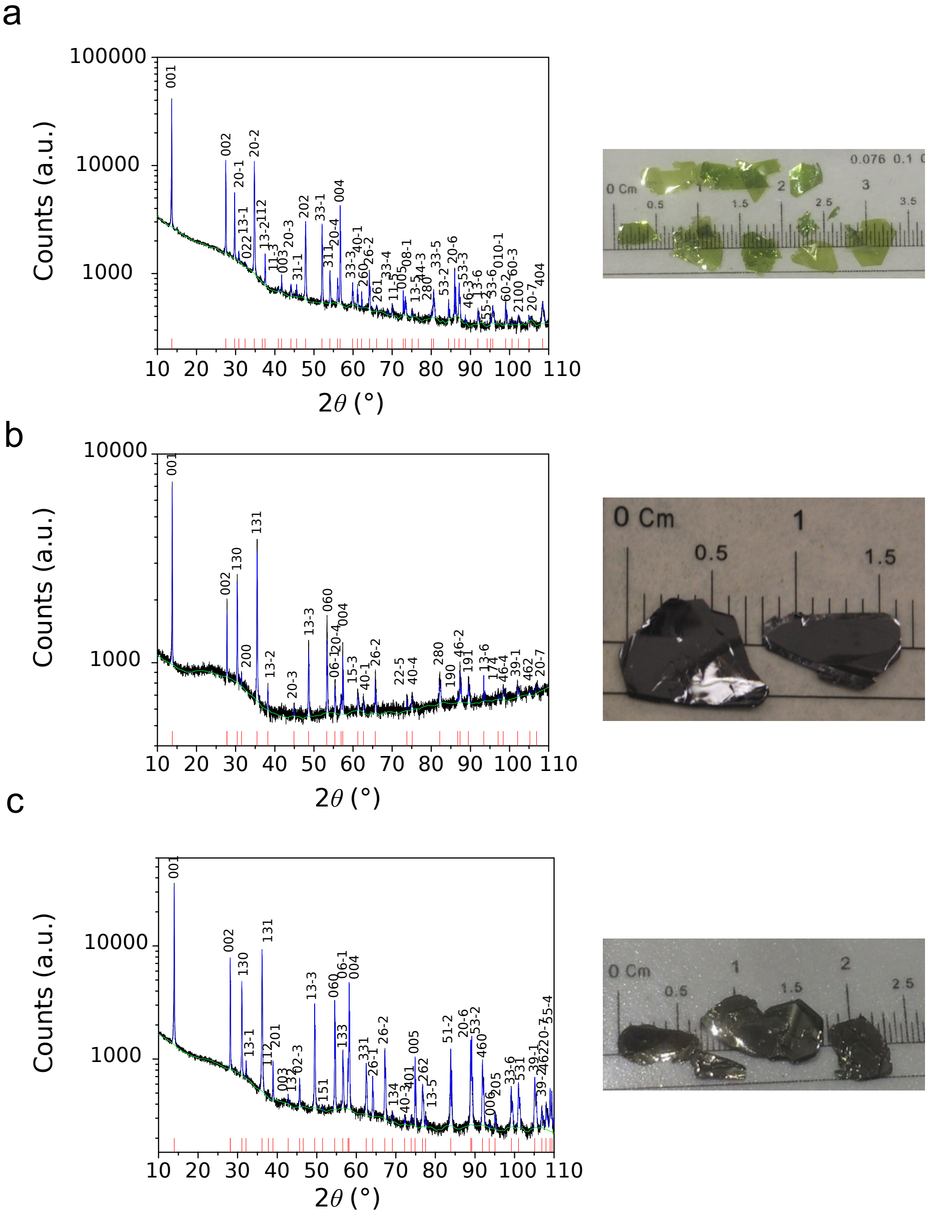

SUPPLEMENTARY FIG. 7. XRPD experimental patterns (black) and corresponding fits (peaks in blue and background in green) for MPS_3 (M = Mn, Fe and Ni) crystals. For clarity, the peaks have been marked by red lines. (a) Left: Pattern of $MnPS_3$. Right: Photography of a $MnPS_3$ crystal. The fit gives as a result: a = 6.077(7) Å, b = 10.55(2) Å and c = 6.805(9) Å, $\alpha = \gamma = 90°$ and $\beta = 107.33(1)°$, monoclinic C face center crystal system with C12/m1 space group, $\chi^2 = 5.8 \times 10^{-5}$. (b) Left: Pattern of $FePS_3$. Right: Photography of a $FePS_3$ crystal. The fit gives as a result: $a = 5.939(6)$ Å, $b = 10.296(3)$ Å and $c = 6.716(3)$ Å, $\alpha = \gamma = 90°$ and $\beta = 107.13(1)°$, monoclinic C face center crystal system with C12/m1 space group, $\chi^2 = 1.5 \times 10^{-6}$. (c) Left: Pattern of $NiPS_3$. Right: Photography of a $NiPS_3$ crystal. The fit gives as a result: $a = 5.815(4)$ Å, $b = 10.087(5)$ Å and $c = 6.627(4)$ Å, $\alpha = \gamma = 90°$ and $\beta = 106.945(9)°$, monoclinic C face center crystal system with C12/m1 space group, $\chi^2 = 1.1 \times 10^{-5}$.

For obtaining large crystals, 4 mmol of the previous material was mixed with I_2 as a transport agent ($[I_2] \sim 5$ mg cm^{-3}) in an evacuated quartz ampoule ($P \sim 5 \times 10^{-5}$ mbar, length = 50 cm, internal diameter = 1.5 cm). The quartz tube was placed inside a three-zone furnace with the material in the leftmost zone. The other two zones were heated up in 24 h from room temperature to 650 °C and kept at that temperature for one day. After this, the leftmost side was heated up to 700 °C in 3 h and a gradient of 700 °C/650 °C/675 °C was established in the three-zone furnace. Then the temperature was kept constant for 28 days and cooled down naturally. As shown in Supplementary Fig. 7, with this process we could obtain crystals with a length up to several centimeters. The obtained crystals were analyzed by ICP-OES (Inductively Coupled Plasma - Optical Emission Spectrometry) and powder X-ray diffraction. The relative weights of elements obtained are summarized in Supplementary table 1. The refinement of the X-ray diffraction pattern (Supplementary Fig. 7) revealed a monoclinic base-centered crystal system with C12/m1 space group and a unit cell determined by $\alpha = \gamma = 90°$ and $\beta = 107.33(1)°$ ($MnPS_3$), $\beta = 107.13(1)°$ ($FePS_3$), $\beta = 106.945(9)°$ ($NiPS_3$), and $a = 6.077(7)$ Å, $b = 10.55(2)$ Å and $c = 6.805(9)$ Å for $MnPS_3$, $a = 5.939(6)$ Å, $b = 10.296(3)$ Å and $c = 6.716(3)$ Å for $FePS_3$ and $a = 5.815(4)$ Å, $b = 10.087(5)$ Å and $c = 6.627(4)$ Å for $NiPS_3$. The obtained results are in accordance with the ones reported in the literature [25].

The crystal growth and characterization of 2H-TaS_2 was performed as already reported in earlier works [26, 27].

**Element**		**Obtained (%)**	**Expected (%)**
**$MnPS_3$**	**Mn**	28.0 ± 1.0	30.2
	**P**	12 ± 1	17.0
	**S**	49 ± 2	52.8
**$FePS_3$**	**Fe**	30.0 ± 1.0	30.5
	**P**	15.7 ± 0.5	16.9
	**S**	51 ± 2	52.6
**$NiPS_3$**	**Ni**	30.0 ± 1.0	31.6
	**P**	15.7 ± 0.5	16.6
	**S**	53 ± 2	51.8

SUPPLEMENTARY TABLE I. Experimental and expected relative weights analyzed by inductively coupled plasma - optical emission spectrometry (ICP-OES) for the different MPS_3 crystals (M = Mn, Fe and Ni).

Supplementary Note 8. REPRODUCIBILITY OF MEASUREMENTS

In order to assess reproducibility of the measured effect between the samples, we show the Q^{-1} data for the $FePS_3$ sample from Fig. 2 of the main text (Drum 1) compared with the data for one additional sample (Drum 2) in Supplementary Fig. 8.

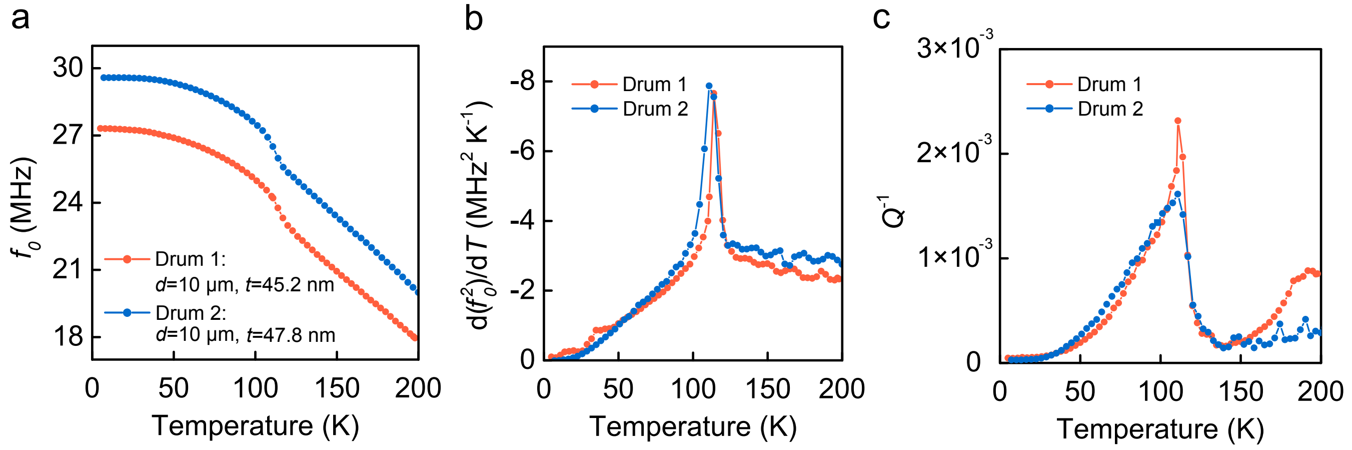

SUPPLEMENTARY FIG. 8. Mechanical properties of a $FePS_3$ resonators with membrane thicknesses indicated in a legend. (a) Resonance frequency as a function of temperature. (b) Temperature derivative of f_0^2 for both samples. (c) Measured mechanical damping Q^{-1} as a function temperature.

We choose the sample parameters (i.e., thickness, t, and drum diameter, d) to be similar for a fair comparison. The features related to phase transition in both frequency (Supplementary Fig. 8b) and quality factor (Supplementary Fig. 8c) are present in both cases. The drum 2, however, has a higher pre-stress accumulated in a result of the fabrication, which is concluded from a notable frequency mismatch in Supplementary Fig. 8a. Some variation in $T_{\rm N}$ of around $2-3$ K can be noted between the samples in Supplementary Fig. 8b, which we attribute to the pre-strain variation in a result of fabrication since, as we report in Fig. 3 of the main text, $T^*_{\rm N}$ of $FePS_3$ is very sensitive to strain. In Supplementary figure 9, the Q^{-1} terms from Supplementary Fig. 8c are displayed and compared to $c_{\rm v}\times T$ terms for both drums. We note that the position of the peak for Q^{-1} and $c_{\rm v}\times T$ corresponds to each other up to the resolution of the measurement.

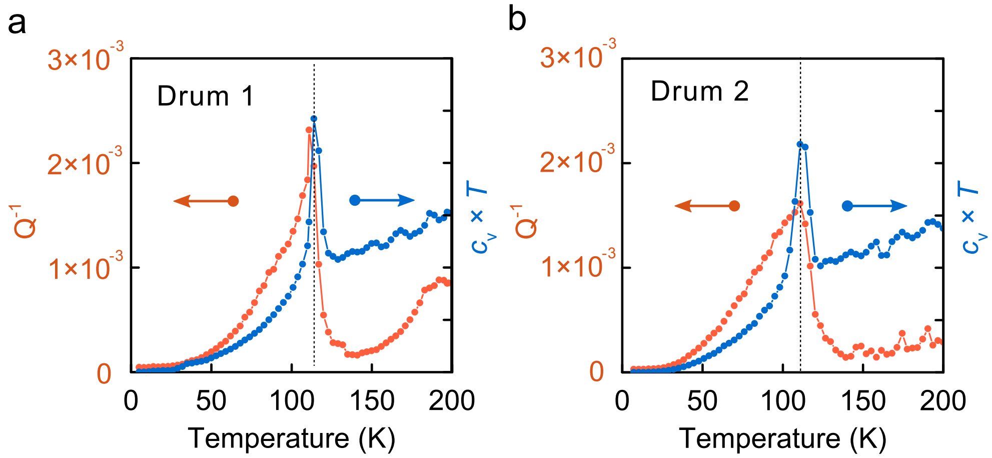

SUPPLEMENTARY FIG. 9. Measured mechanical damping $Q^{-1}(T)$ as a function temperature in filled orange dots in both panels for samples form Supplementary Fig. 8. Filled blue dots in both panels - normalized $c_{\rm v}(T)\ T$ term (see Supplementary Note 4).

The results for $FePS_3$ are also well reproducible within a single device (Drum 2) with no hysteresis observed in both frequency and Q-factor for multiple temperature sweeps, as shown in Supplementary Fig. 10.

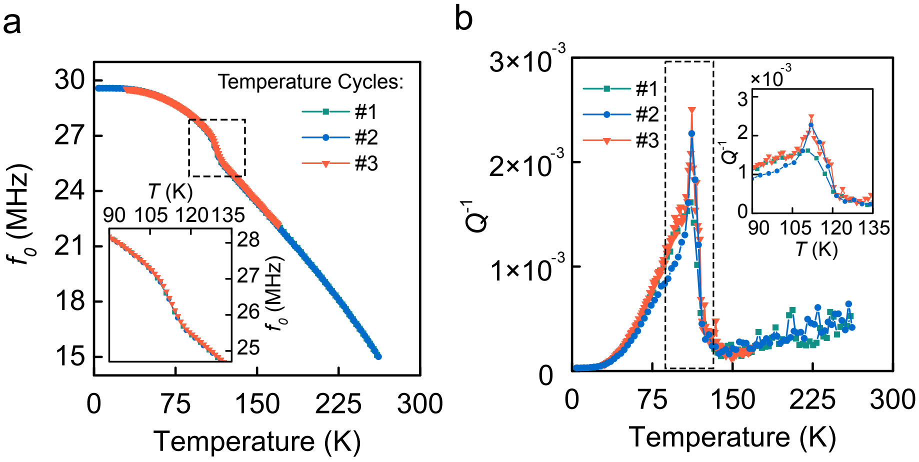

SUPPLEMENTARY FIG. 10. Multiple temperature cycles for Drum 2 from Supplementary Fig. 8. (a) Filled dots - resonance frequency as a function of temperature. Inset: a close-up of the region indicated with dashed line box. (b) Filled dots - measured mechanical damping Q^{-1} as a function temperature. Inset: a close-up of the region indicated with dashed line box.

[1] Wah, T. Vibration of circular plates. *J. Acoust. Soc. Am.* **34**, 275–281 (1962).
[2] Castellanos-Gomez, A. *et al.* Single-layer MoS_2 mechanical resonators. *Adv. Mater.* **25**, 6719–6723 (2013).
[3] Timoshenko, S., Young, D. H. & Weaver, W. *Vibration Problems in Engineering* (Wiley, New York, 1974), 4 edn.
[4] Singh, V. *et al.* Probing thermal expansion of graphene and modal dispersion at low-temperature using graphene nano-electromechanical systems resonators. *Nanotechnology* **21**, 165204 (2010).
[5] Morell, N. *et al.* High quality factor mechanical resonators based on WSe_2 monolayers. *Nano Lett.* **16**, 5102–5108 (2016).
[6] White, G., Birch, J. & Manghnani, M. H. Thermal properties of sodium silicate glasses at low temperatures. *J. Non-Cryst. Solids* **23**, 99–110 (1977).
[7] Lyon, K. G., Salinger, G. L., Swenson, C. A. & White, G. K. Linear thermal expansion measurements on silicon from 6 to 340 K. *J. Appl. Phys.* **48**, 865–868 (1977).
[8] Takano, Y. *et al.* Magnetic properties and specific heat of MPS_3 (M=Mn, Fe, Zn). *J. Magn. Magn. Mater.* **272-276**, E593–E595 (2004).
[9] Kim, K. *et al.* Suppression of magnetic ordering in XXZ-type antiferromagnetic monolayer $NiPS_3$. *Nat. Commun.* **10** (2019).
[10] Sanditov, D. S., Mashanov, A. A., Darmaev, M. V., Sanditov, B. D. & Mantatov, V. V. Grüneisen parameter and elastic constants of crystals and vitreous bodies. *Russ. Phys. J.* **52**, 221–230 (2009).
[11] Belomestnykh, V. N. & Tesleva, E. P. Interrelation between anharmonicity and lateral strain in quasi-isotropic polycrystalline solids. *Tech. Phys.* **49**, 1098–1100 (2004).
[12] Hashemi, A., Komsa, H.-P., Puska, M. & Krasheninnikov, A. V. Vibrational properties of metal phosphorus trichalcogenides from first-principles calculations. *J. Phys. Chem. C* **121**, 27207–27217 (2017).
[13] Zhang, X., Zhao, X., Wu, D., Jing, Y. & Zhou, Z. $MnPSe_3$ monolayer: A promising 2D visible-light photohydrolytic catalyst with high carrier mobility. *Adv. Sci.* **3**, 1600062 (2016).
[14] Joy, P. A. & Vasudevan, S. Magnetism in the layered transition-metal thiophosphates MPS_3 (M=Mn, Fe, and Ni). *Phys. Rev. B* **46**, 5425–5433 (1992).
[15] Landau, L. D. On the theory of phase transitions. *Zh. Eksp. Teor. Fiz.* **7**, 19–32 (1937). [*Ukr. J. Phys.* **53**, 25 (2008)].
[16] Landau, L. D., Pitaevskii, L. P. & Lifshitz, E. M. *Electrodynamics of continuous media*, vol. 8 (Butterworth, New York, 1984), 2 edn.
[17] Schmid, S., Villanueva, L. G. & Roukes, M. L. *Fundamentals of Nanomechanical Resonators* (Springer International Publishing, 2016).
[18] Zener, C. Internal friction in solids. I. Theory of internal friction in reeds. *Phys. Rev.* **52**, 230–235 (1937).
[19] Zener, C. Internal friction in solids II. General theory of thermoelastic internal friction. *Phys. Rev.* **53**, 90–99 (1938).
[20] Lifshitz, R. & Roukes, M. L. Thermoelastic damping in micro- and nanomechanical systems. *Phys. Rev. B* **61**, 5600–5609 (2000).
[21] Small, M. K. & Nix, W. Analysis of the accuracy of the bulge test in determining the mechanical properties of thin films. *J. Mater. Res. Technol.* **7**, 1553–1563 (1992).
[22] Weber, P., Güttinger, J., Tsioutsios, I., Chang, D. E. & Bachtold, A. Coupling graphene mechanical resonators to superconducting microwave cavities. *Nano Lett.* **14**, 2854–2860 (2014).
[23] Jiang, J.-W. & Zhou, Y.-P. Parameterization of Stillinger-Weber potential for two-dimensional atomic crystals. In *Handbook of Stillinger-Weber Potential Parameters for Two-Dimensional Atomic Crystals* (IntechOpen, 2017).
[24] Abdel-Hafiez, M. *et al.* Enhancement of superconductivity under pressure and the magnetic phase diagram of tantalum disulfide single crystals. *Sci. Rep.* **6** (2016).
[25] Ouvrard, G., Brec, R. & Rouxel, J. Structural determination of some MPS_3 layered phases (M = Mn, Fe, Co, Ni and Cd). *Mater. Res. Bull.* **20**, 1181–1189 (1985).
[26] Pinilla-Cienfuegos, E. *et al.* Local oxidation nanolithography on metallic transition metal dichalcogenides surfaces. *Appl. Sci.* **6**, 250 (2016).
[27] Navarro-Moratalla, E. *et al.* Enhanced superconductivity in atomically thin TaS_2. *Nat. Commun.* **7** (2016).
